# Improving patient-centered communication in breast cancer: a study protocol for a multilevel intervention of a shared treatment deliberation system (SharES) within the NCI community oncology research program (NCORP) (Alliance A231901CD)

**DOI:** 10.1186/s13063-022-07048-4

**Published:** 2023-01-06

**Authors:** Sarah T. Hawley, Kelley Kidwell, David Zahrieh, Anne McCarthy, Rachel Wills, Aaron Rankin, Timothy Hofer, Selina Chow, Reshma Jagsi, Heather Neuman

**Affiliations:** 1 Department of Medicine, University of Michigan, Ann Arbor, MI USA; 2 Ann Arbor VA Center for Clinical Management Research, Ann Arbor, MI USA; 3 Rogel Cancer Center, University of Michigan, Ann Arbor, MI USA; 4 School of Public Health, Department of Biostatistics, University of Michigan, Ann Arbor, MI USA; 5 Department of Quantitative Health Sciences and Alliance Statistics and Data Management Center (SMDC), Mayo Clinic, Rochester, MN USA; 6 Ultragenyx Pharmaceutical, Novato, CA USA; 7 American College of Surgeons, Chicago, IL USA; 8 Alliance Protocol Operations Office, Chicago, IL USA; 9 Department of Radiation Oncology, University of Michigan, Ann Arbor, MI USA; 10 University of Wisconsin School of Medicine and Public Health, Madison, WI USA

**Keywords:** Breast cancer, Decision-making, Communication, Cancer care delivery, Trial

## Abstract

**Background:**

Advances in precision medicine have given oncologists new evaluative tools to better individualize treatments for patients with curable breast cancer. These innovations have revealed a need to improve patient understanding of novel, often complex information related to breast cancer treatment decisions. Ensuring patients have the emotional support to face consequential treatment decisions, as well as the opportunity to engage and discuss with their clinicians, is key to improving patient-centered communication and patient understanding.

**Methods/design:**

This study will implement a multilevel intervention with patient and clinician components as a NCORP Cancer Care Delivery Research (CCDR) trial within the Alliance for Clinical Trials in Oncology Research Base (Alliance). The two interventions in this study, the *Shar*ed Decision *E*ngagement *S*ystem (SharES), include (1) two versions of an evidence-based patient-facing breast cancer treatment decision tool (iCanDecide +/− an emotional support module) and (2) a clinician-facing dashboard (Clinician Dashboard) that is reviewed by surgeons/clinicians and summarizes ongoing patient needs. The design is a near minimax, hybrid stepped wedge trial of SharES where both interventions are being evaluated in a crossed design over six 12-week time periods. The primary outcome (knowledge) and key secondary outcomes (i.e., self-efficacy and cancer worry) are assessed via patient report at 5 weeks after surgery. Secondary outcomes are also assessed at 5 weeks after surgery, as well as in a second survey 9 months after registration. We anticipate recruiting a total of 700 breast cancer patients (600 evaluable after attrition) from 25 surgical practices affiliated with Alliance.

**Discussion:**

Upon study completion, we will have better understanding of the impact of a multilevel intervention on patient-centered communication in breast cancer with a specific focus on whether the intervention components improve knowledge and self-efficacy and reduce cancer worry.

**Trial registration:**

ClinicalTrials.govNCT04549571. Registered on 16 September 2020.

**Supplementary Information:**

The online version contains supplementary material available at 10.1186/s13063-022-07048-4.

## Administrative information

Note: the numbers in curly brackets in this protocol refer to SPIRIT checklist item numbers. The order of the items has been modified to group similar items (see http://www.equator-network.org/reporting-guidelines/spirit-2013-statement-defining-standard-protocol-items-for-clinical-trials/).

**Table:** 

Title {1}	Improving Patient-Centered Communication in Breast Cancer: A Study Protocol for a Multilevel Intervention of a Shared Treatment Deliberation System (SharES) within the NCI Community Oncology Research Program (NCORP) (Alliance A231901CD)
Trial registration {2a and 2b}.	NCT04549571
Protocol version {3}	1 September 2022
Funding {4}	This work is supported by the NIH NCI (R01CA R01 CA237046), Principle Investigators (mPIs) Hawley, Jagsi Because this is a Cancer Care Delivery study within the NCI NCORP program, some work is also supported by the following grants to the Alliance U10CA180821, U10CA180882 (SDC) UG1CA189823 (NCORP Research Base).
Author details {5a}	1. 1 Department of Medicine, University of Michigan, Ann Arbor, MI 2. Ann Arbor VA Center for Clinical Management Research, Ann Arbor, MI 3. Rogel Cancer Center, University of Michigan, Ann Arbor, MI 4. School of Public Health, Department of Biostatistics, University of Michigan, Ann Arbor, MI 5. Department of Quantitative Health Sciences and Alliance Statistics and Data Management Center (SMDC), Mayo Clinic, Rochester, MN and 6. Ultragenyx Pharmaceutical, Novato, CA 7. American College of Surgeons, Chicago, IL 8. Alliance Protocol Operations Office, Chicago, IL 9. Department of Radiation Oncology, University of Michigan, Ann Arbor, MI 10. University of Wisconsin School of Medicine and Public Health, Madison, WI
Name and contact information for the trial sponsor {5b}	**NIH/NCI Program Official**: Sarah Kobrin, PhD MPH, Brach Chief, Health Systems and Interventions, NCI: kobrins@mail.nih.gov
Role of sponsor {5c}	The sponsor played no part in study design; collection, management, analysis, and interpretation of data; writing of the report; and the decision to submit the report for publication. The content is solely the responsibility of the authors and does not necessarily represent the official views of the National Institutes of Health or National Cancer Institute.

## Introduction

### Background and rationale {6a}

Advances in precision medicine have given oncologists new evaluative tools to better individualize treatments for patients with curable breast cancer. These innovations have revealed a need to improve patient understanding of novel, often complex, information related to breast cancer treatment decisions. Pressure to make treatment decisions after diagnosis triggers powerful emotional reactions to the disease threat. The National Coalition for Cancer Survivorship suggests that a diagnosis of cancer will create a “…state of crisis in nearly all individuals,” magnified by the fact that most patients are ill-prepared for the emotional and cognitive demands associated with their diagnosis [1, 2]. In this context, advances in precision medicine cannot optimally improve health without parallel progress in patient-centered communication (PCC).

#### Patients need help to manage the complexity of information

Although having an accurate understanding about treatment trade-offs is a key component of an informed decision, patients’ knowledge is surprisingly low. In part, this is due to the complex and often overwhelming nature of the clinical information and treatment decisions a patient must make. Several breast cancer decision tools aimed exclusively at the patient have been developed and tested. In general, these patient-facing tools have been modestly successful at improving knowledge about treatment options. Indeed, our patient-facing decision tool (“iCanDecide”) was effective in improving knowledge about complex treatment tradeoffs using an innovative “knowledge building” approach. We systematically built knowledge by first educating patients with very simple information about key facts related to their treatment; we found that 60% of patients in the intervention arm had high knowledge (defined as at least 80% of knowledge items correct) 4 weeks after enrollment and treatment, compared with 42% who viewed a static version without knowledge building (*P* < 0.001) [3]. Importantly, knowledge deficits are especially concerning in more vulnerable groups, such as those with lower educational attainment and racial/ethnic minorities (particularly those who are non-native English speakers) [4, 5]. We believe this knowledge building approach should be the standard of care for educating patients, and particularly for those most vulnerable to low knowledge where gains are most needed. However, with patient input, we identified two additional strategies that merit inclusion in patient decision support tools: (1) supporting the emotional aspects of diagnosis and (2) more directly engaging clinicians in the process of supporting shared decision-making.

#### Tools need to address and support patients’ emotional needs

The powerful role of emotion in decision-making has been well documented. This stems from the fact that decision-making at its core includes both emotional and rational processes [6, 7]. Moreover, failure to acknowledge emotion in decision-making may lead to suboptimal decisions that, while anchored in facts, may not be aligned with the individual’s sense of self. Two underlying mechanisms for how emotions may influence medical decision-making are particularly important for this study. First, psychological processes, such as affective forecasting (i.e., trying to predict how one will feel in the future, leading to making decisions to avoid regretting something in the future) and catastrophizing (i.e., weighing what might happen) can lead to quick decisions favoring more extensive treatment that may not be medically necessary, and bias decisions away from patients’ underlying values [8, 9]. Second, anxiety, distress, and worry—always present with a cancer diagnosis—can make it difficult to accurately comprehend risk and benefit information. This is especially true in certain groups where knowledge deficits are biggest, such as those of lower educational attainment and racial/ethnic minorities [4–6, 10, 11]. Worry contributes to feelings of lack of control and has been associated with choices for extensive treatment, reduced quality of life, financial toxicity, and distress [12–14]. We have found that when faced with a cancer diagnosis, many patients express a default preference to “get the cancer out” and an urgency for surgical treatment (e.g., to “achieve peace of mind”) with low knowledge about the risks and benefits. This combination of worry and low knowledge can result in choices for extensive treatment. Tools that address these underlying emotional drivers can allow patients to better process cognitive information and manage their worry, potentially helping them realize peace of mind with less extensive options.

#### Tools need to help clinicians identify their patients’ cognitive and emotional needs

A key gap in existing decision tools is that they have been developed for stand-alone use by a patient. Thus, the very tools designed to support shared decision-making place the onus on patients to bring up what they have learned from the tool with their clinicians. Yet, patients are often unaware of the gaps they have in their knowledge or about how their emotions may be influencing their treatment choices. Research has identified the need to provide clinicians with feedback regarding continued threats to poor decision-making, but to date, no patient decision tools have been integrated within a system to do this. Further, clinicians do not have tools that capture both cognitive and emotional needs of their patients. With pilot funding from our cancer center, our team developed and is testing a Clinician Dashboard that feeds back the patient experience with our current version of iCanDecide, including remaining knowledge deficits, values for treatment options, degree of anxiety and distress, and assessment of patient-centered and overall communication with their clinicians. The Clinician Dashboard allows clinicians to identify patients who need follow-up, or “circling back” with the goal of addressing these deficits and supporting patient-clinician communication.

#### Shared tools are needed to support patient-centered communication (PCC)

While our prior work showed the promise of iCanDecide, it revealed the need to broaden the concept of decision quality to focus on improving key aspects of PCC. Improving the process of decision-making and conceptualization of decision quality may ultimately lead patients to feel more comfortable making less extensive, but equally effective, treatment choices. Doing so may also translate to longer term positive outcomes for patients, such as quality of life (QoL) and financial outcomes [15, 16]. Patients and clinicians need to be empowered to manage both the cognitive and emotional aspects of, and to share, these decisions. To this end, we propose to formally evaluate a Shared Decision Engagement System (SharES) that integrates the patient-facing, emotional support enhanced iCanDecide tool with a Clinician Dashboard. By partnering with the Alliance NCORP Research Base within the NCI Community Oncology Research Program (NCORP), we will be well positioned not only for successful completion of the proposed trial but for scalability and dissemination throughout the NCORP network.

#### Conceptual framework

**Fig. 1:**
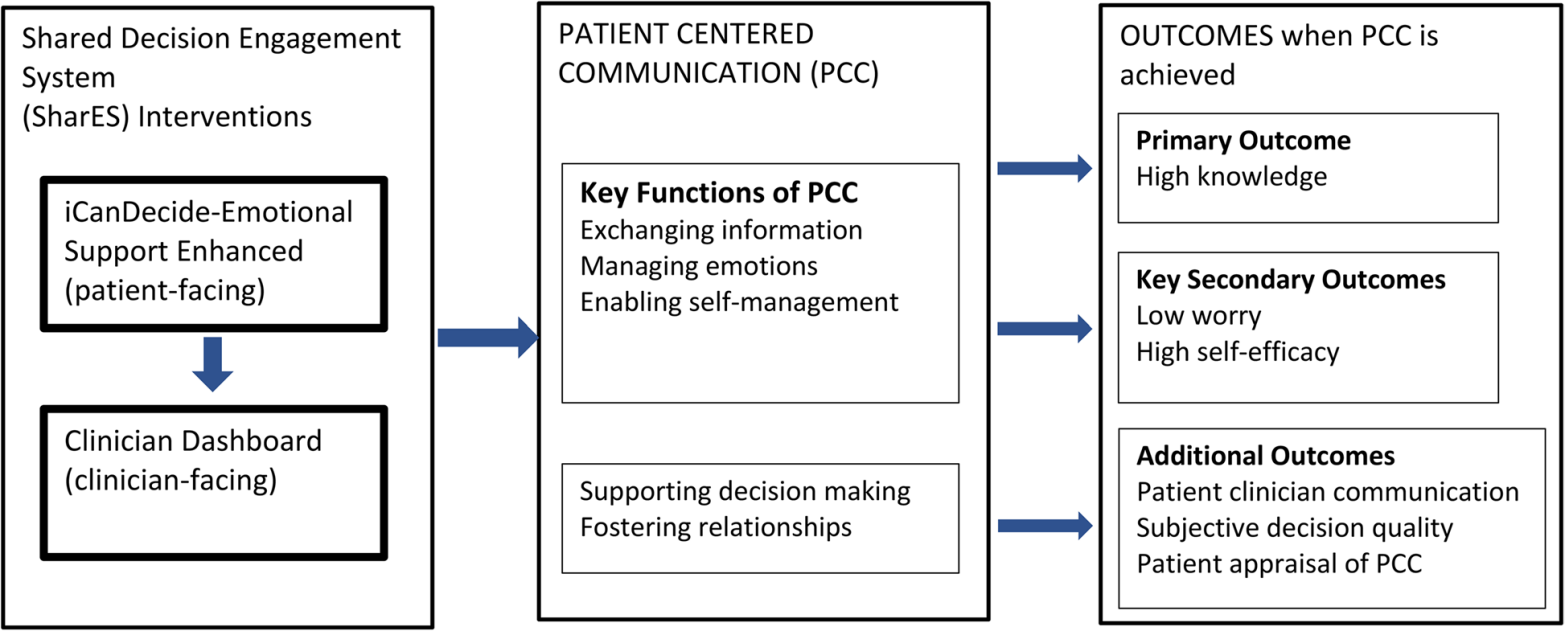
Conceptual framework: overview of how SharES will impact patient-centered communication and outcomes

Our conceptual framework (Fig. 1) describes how SharES will impact key components of PCC and expected outcomes. Our framework is informed by Lerner’s framework for the role of emotion in decision making, Feldman-Stewart’s model for patient-professional communication, and our preliminary studies [11, 17]. SharES will directly address three core focus areas that are aligned with the NCI’s components of PCC and areas that are not well met by existing decision tools. This will be done through enhancements to iCanDecide as well as through Clinician Dashboard features. Patients’ cognitive processing of information will be better through the support of patients’ response to emotion and enable them to manage their cancer-related worry. These primary focus areas will translate to improvements in knowledge, the primary outcome of the SharES trial, as well as renewed self-efficacy and reduced worry, the secondary outcomes. By addressing these areas, SharES will support overall decision-making and relationship building, which will be assessed through the secondary outcomes of global constructs of PCC, specifically patient-clinician communication (the Health Care Climate questionnaire), Subjective Decision Quality (scale developed by our team in prior studies assessing patient-reported domains of satisfaction with information, involvement, time, process and regret with decision-making), and Patient Assessment of Cancer Communication Experiences (PACE), and values concordant treatment. We hypothesize that both components of SharES will have direct additive effects on these components of PCC. We will further assess the perception of the components of SharES from the perspective of patients and clinicians in our process evaluation.

This study will address two key gaps related to improving decision quality and outcomes in breast cancer and improve PCC: (1) helping manage the flood of patient emotional responses to a breast cancer diagnosis and (2) closing the PCC loop by better connecting clinicians to the patient experience with the tool. Therefore, we will evaluate an emotional support enhanced version of iCanDecide-ESE (vs. standard iCanDecide-S) and integrate it into breast oncology workflow by linking patient feedback to a Clinician Dashboard. We will evaluate the impact of this shared decision engagement system (SharES) on knowledge, as well as key secondary patient-reported outcomes related to cancer worry and distress, and other outcomes related to patient centered communication.

### Objectives {7}

The overall goal of this study is to evaluate the impact of a multilevel intervention containing two concurrent interventions on patient-centered communication outcomes in breast cancer patients. The interventions being evaluated are (1) a patient-level comparison of an evidence-based breast cancer treatment decision tool called iCanDecide (the Standard [S] version vs. a version with an emotional support enhancement [ESE]) and (2) a clinician-level dashboard providing clinicians information about lingering patient needs after viewing the decision tool. We have two primary co-objectives:

1. To demonstrate that the emotional support enhanced iCanDecide (iCanDecide-ESE) intervention is more effective than the standard version (iCanDecide-S), resulting in higher patient knowledge of treatment risks and benefits.
2. To demonstrate that the activation of the Clinician Dashboard is more effective than not using a Clinician Dashboard, resulting in higher patient knowledge of treatment risks and benefits.

### Trial design {8}

**Fig. 2:**
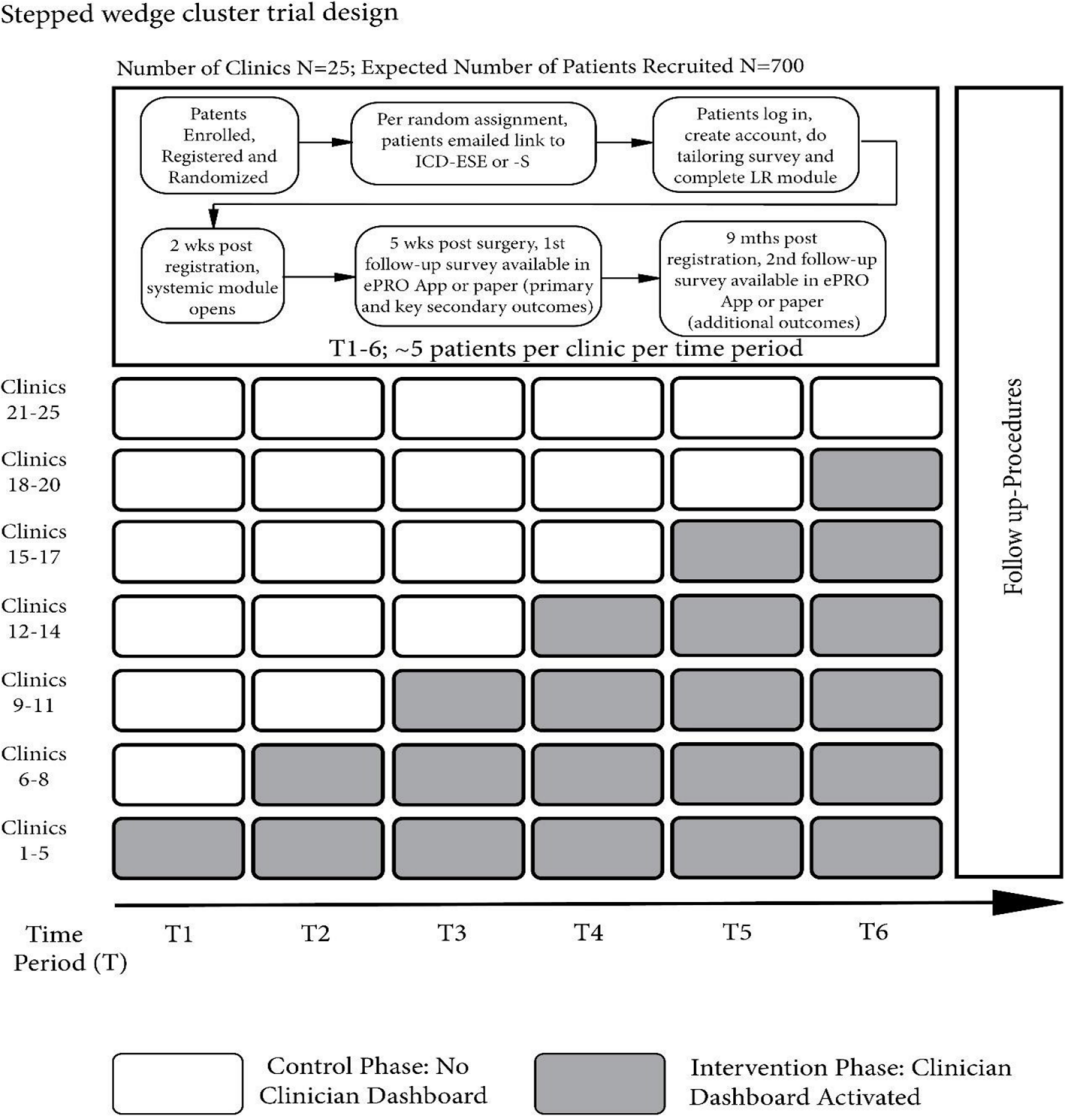
Stepped wedge design and timeline

This study is a multilevel, near minimax, hybrid stepped wedge trial that has two interventions being evaluated in a crossed design over six 12-week time periods. Primary and secondary outcomes are obtained from patient report 5 weeks after surgery to evaluate both components of SharES (i.e., patient facing iCanDecide and the Clinician Dashboard) in 600 evaluable patients from 25 surgical practices (Fig. 2). The study will take 5 years, with trial field work taking approximately 22 months.

## Methods

### Setting {9}

SharES will be conducted as a Cancer Care Delivery Research (CCDR) study within the National Cancer Institute’s (NCI) Community Oncology Research Program (NCORP). The NCORP primary Research Base for this study is the Alliance for Clinical Trials in Oncology (Alliance). This trial is sponsored by the Alliance/American College of Surgeons CCDR Committee. Funding for the study is available to NCORP sites with CCDR-restricted funds. SharES will be conducted in 25 separate surgical clinics/practices affiliated with the Alliance, as well as with other NCORP Research Bases (e.g., SWOG, ECOG-ACRIN). Three of the 25 participating surgical practices will be from NCORP-designated minority/underserved community sites. Sites participating in SharES will receive two Alliance membership accrual credits.

### Eligibility criteria {10}

SharES has three levels of participants, including surgical clinics, clinicians (breast surgeons or advanced practice providers helping care for breast cancer patients), and newly diagnosed breast cancer patients. Criteria for each is described below.

#### Surgical clinics

Eligible clinics will be NCORP-affiliated surgical practices that are rostered and funded components of the NCORP institutions. They are surgical clinics where initial locoregional treatment for breast surgery decision-making is done. Twenty-five surgical clinics will be recruited to participate across NCORP to obtain a representative and diverse cohort of patients. Additional practice eligibility requirements include the following: (1) clinics must annually provide surgical care for over 100 patients newly diagnosed breast cancer patients and (2) clinics must have at least one surgeon who does breast surgery who agrees to participate in the study. These requirements necessitate significant communication between the study team and the clinics to determine which ones have the capacity to participate throughout the study and meet accrual goals. NCORP surgical clinics enrolled in SharES may accept a $1000 incentive following enrollment of the first patient. They may also accept provision of loaner iPads in cases where the patient population may have limited technologic access. Both the incentive and iPads will be charged back to the overall parent grant (R01 CA237046).

#### Clinicians

Clinicians eligible for SharES include practicing breast surgeons and their designee(s) (e.g., physician assistants (PA), nurse practitioners, clinical nurse specialists, or nurses) that participate in the treatment decision-making process with breast cancer patients. At least one surgeon at each clinic must consent to participate in the study. Surgeons must be rostered with the NCI to participate. S/he may identify a nurse, PA, or advanced practice provider with whom s/he works that is involved in the delivery of the care of the same patients to participate. Clinicians must agree to have their patients recruited for the entire time the study is open at their clinic, which will include time periods in which the clinicians will—and time periods in which they will not—have access to the Clinician Dashboard (see Fig. 2).

#### Patients

Patients eligible for SharES must have a new diagnosis of early stage (0–III) breast cancer, aged 21–84 years, and able to speak English or Spanish. Eligible patients must be planning breast surgery as a component of their definitive treatment (breast conservation or mastectomy) and must be receiving care from a clinician who has consented to participate in the study.

### Who will take informed consent? {26a}

Informed consent will be taken by trained clinical research assistants (CRAs) and/or clinicians at each of the 25 participating surgical clinics. Those taking informed consent have experience doing so from participation in NCORP, for both treatment and CCDR trials. They will take consent from patient and clinician participants, after which participants will be formally enrolled and registered into the Alliance OPEN registration system. Facilitated remote consent is optional.

### Additional consent provisions for collection and use of participant data and biological specimens {26b}

Not applicable.

## Interventions

### Explanation for choice of comparators {6b}

There are two interventions being tested in SharES. This study will cross a patient-level randomization of an emotional support enhanced version of the iCanDecide breast cancer treatment decision tool (iCanDecide-ESE) vs. standard iCanDecide (iCanDecide-S) with a hybrid stepped wedge trial of a Clinician Dashboard to promote patient-centered communication. All patients enrolled will receive access to one of the patient interventions that will be delivered throughout the study, while clinics will receive access to the Clinician Dashboard in a hybrid stepped wedge format, with some getting access the entire time, others never getting access, and others getting access starting at various times (or “steps”). The choice of comparators for both levels is provided in the “Background and rationale {6a}” section. Briefly, we believe it is important to compare an evidence-based, standard patient-facing tool to one with emotional support to improve PCC. We need to close the loop by providing clinicians access and insight into patients with lingering deficits via the Clinician Dashboard so as to compare to standard of care where clinicians do not have access to this information.

### Intervention descriptions {11a}

Both interventions in SharES are digital interventions developed by the study team in collaboration with the University of Michigan (UM) Center for Health Communications Research (CHCR), a technology development team with over 20 years of experience developing web-based, digital, and mobile interventions. The patient-facing tool can be viewed on a mobile or tablet device, while the Clinician Dashboard is best viewed in a laptop computer because of how the intervention information is displayed. Both interventions use secure log-in systems where participants create a username and password. Both have been vetted by the UM Information Assurance (IA) office that sets standards for digital interventions being used or deployed from UM.

#### Patient-facing iCanDecide decision tool

The standard iCanDecide decision support tool (iCanDecide-S) was previously developed by members of the study team, tested in a large randomized trial (vs. standard online information) and proven effective for improving knowledge about breast cancer and patient-preparedness related to treatment decision making [3]. SharES will test an “emotional support enhanced” version of the standard iCanDecide (iCanDecide-ESE). The previously tested version is being used as the control, or “standard” (S) version for this study. Each version of iCanDecide has five sections corresponding to an expanded version of Elwyn’s shared decision-making model (connect, choice, options, decision, and summary) [18]. The iCanDecide-S version has been updated to include new content related to breast cancer treatment, including genetic testing. The iCanDecide-ESE version includes all the content delivered in the S version but has additional features that support the key functions of PCC outlined in the conceptual framework (Fig. 1) for each of the sections. These include two modules specifically designed to address the emotions, worry, and distress associated with a breast cancer diagnosis: (1) expectation management and (2) worry/emotional support. Each of these new sections includes established methods for emotional regulation and support (e.g., cognitive behavioral therapy, use of norm reinforcement, mindfulness). Users of both versions move through sections in a linear way. Both versions populate the same information into the Clinician Dashboard that is viewable by surgeons/clinicians at participating clinics (when randomized to their step). Patients may create PDF versions of any sections of either version of the tool that are printable and able to be shared with other cancer clinicians (medical, radiation oncologists) or family members. The tool is available in English or Spanish, and participants choose desired language when they log in. Because both versions of the tool are called “iCanDecide”, patients are blinded to whether they are in the S or ESE arm. Clinicians and clinics are also blinded as to which arm the patient is assigned.

#### Clinician-facing dashboard

The Clinician Dashboard is a web platform viewable by clinicians (surgeons or designee) that populates with information following an enrolled patient’s use of iCanDecide. The Clinician Dashboard provides clinicians an online platform that summarizes their patients’ (1) lingering knowledge deficits (incorrect items on the selected knowledge questions), (2) values that are misaligned with planned treatment, and (3) an indicator of the emotional state of the patient (score ≥ 4 on the distress thermometer, determined to be clinically meaningful anxiety), and reasons for it. It also offers a write-in for unanswered questions. The Clinician Dashboard is configured so that it can be used before or after the patient’s first visit (or both) and can be used by surgeons or other clinicians who engage in treatment decision-making. Clinician Dashboard training will be conducted about 2 weeks before the practice begins use of the Clinician Dashboard. After activation of the Clinician Dashboard at each clinic, participating clinicians will receive email alerts when a patient has completed the patient-level intervention. The clinician may then log into the Clinician Dashboard using the secure and password-protected account to view their patients’ reports. These reports will be listed in the Clinician Dashboard starting with the most recent. This process will continue for the duration of the Clinician Dashboard intervention period for that clinic. Clinicians will utilize the Clinician Dashboard in whatever way/amount they find useful.

### Criteria for discontinuing or modifying allocated interventions {11b}

Intervention assignment will not be modified during the study. Participants will remain on study unless they themselves withdraw (for both patient and clinicians), in which case the intervention will no longer be available to them.

### Strategies to improve adherence to interventions {11c}

Each intervention can be used as much or as little as desired by the participant following enrollment (for both patients and clinicians). “Dose” of intervention will be assessed via web-based data on page views, time on page, etc., to understand more about the level of use of each intervention by the participants. Participants are provided incentives to join the study and engage in the follow-up assessments.

### Relevant concomitant care permitted or prohibited during the trial {11d}

Usual care for breast cancer patients will be continued throughout the trial. There is nothing prohibited.

### Provisions for post-trial care {30}

Post-trial, all participants will continue to receive usual care for breast cancer patients.

### Outcome measures {12}

#### Primary outcome

The primary outcome of this study is knowledge about the risks and benefits of locoregional treatment measured at the first follow-up assessment. This measure was previously developed and tested by the study team and is defined as the percentage of correct answers (range: 0–100%) from a 5-item scale: higher percentages indicate higher levels of knowledge about treatment risks and benefits [3].

#### Secondary outcomes

We will assess two *key secondary outcomes*, patient-reported cancer worry and breast cancer self-efficacy, both measured at the first follow-up assessment. Cancer worry is measured via the validated eight-item Cancer Worry Scale [19] assessing the degree of cancer-related worry (range: 8 to 40); with higher scores indicating more frequent worries about cancer. Breast cancer self-efficacy is an 11-item validated scale designed to assess breast cancer patients’ overall feelings of control of their cancer and worry about their cancer [20]. It is the composite score obtained from the 11-item validated scale designed to assess breast cancer patients’ overall feelings of control of their cancer and worry about their cancer (range: 11 to 55), with higher scores indicating increased self-efficacy.

There are four *additional secondary outcomes* for this study that each assess additional aspects of PCC. Each are collected at the first follow-up assessment and measured via existing instruments: (1) the six-item Health Care Climate Questionnaire [21, 22], (2) the 12-item Patient Assessment of Cancer Experiences (PACE) [23], (3) the 5-item subjective decision quality (SDQ) measure [24], and (4) three emotional support measures [14].

#### Exploratory outcomes

At 9 months after registration, patients who had invasive cancer at time of enrollment will be surveyed to assess their decision-making and communication regarding systemic treatment. This survey will include a 5-item measure of knowledge about systemic treatment risks and benefits, used in our prior work [3]. At this assessment, the participant will also complete the Cancer Worry Scale a second time, as well as the distress thermometer and the FACT-B Quality of Life scale [25, 26].

#### Clinician measures

We will conduct assessments at time of registration/enrollment, 9 months and 18 months after clinician registration to assess clinicians’ perceptions of their ability to deliver PCC. The two paper surveys include the Patient-Practitioner Orientation scale (PPOS-18 items) [27] and the clinician version of the Health Care Communication Questionnaire (HCCQ) (six items) [21, 22].

### Participant timeline {13}

See Fig. 2 that outlines the stepped wedge time periods and follow-up assessment points.

### Sample size {14}

Sample size depends on the near minimax stepped wedge/parallel hybrid design as described by Girling and Hemming [28]. This design provides close to the maximal possible efficiency across a range of intra-cluster correlations (ICCs) from 0 to 0.2, where larger ICCs are expected for our primary outcome of knowledge as seen from the prior iCanDecide randomized clinical trial (RCT) [3]. Specifically, in our previous iCanDecide trial, we calculated an ICC of 0.15 and a moderate effect size for the iCanDecide decision tool of 0.5 standard deviations. This effect size is equivalent to a little less than one additional question being answered correctly by those who received the intervention.

For SharES, we calculated the sample size required to detect a somewhat smaller standardized effect size of 0.35, which results in only two out of three women getting an additional question correct. Thus, for the planned hybrid stepped wedge design testing the clinic level intervention, we will recruit 25 clinics with a target total sample size of 600 evaluable patients (approximately four patients per clinic per time period) [28–31]. We estimate about 15% will not complete the first follow-up assessment (5 weeks post-surgery), based on our prior iCanDecide trial [3], so we plan to enroll at least 700 participants or around 5 patients per clinic per period. This will allow us to have at least 80% power (alpha=0.05) to detect differences of 0.35 standard deviations between intervention and control subjects for the clinic level intervention (Clinician Dashboard vs. none) and 0.25 standard deviations for the individual-level intervention (ICanDecide-ESE vs. ICanDecide-S) across a wide range of ICCs (0.01 to 0.20). The interaction effect between the two interventions would have to be at least 0.5 standard deviations, or about one additional question correct for each subject, comparable to our effect size in our current intervention, for us to be able to detect it at conventional significance levels.

### Recruitment {15}

Because of the three types of participants in SharES (clinics, clinicians and patients), different recruitment methods are needed for each type. Each is described below.

Surgical clinics

SharES is a CCDR study and is only available to NCORP-affiliated surgical clinics.

Interested clinics will be recruited via Alliance-sponsored webinars and presentations at Alliance meetings. Interested practices must have at least one breast surgeon who is willing to participate, meaning they must agree to consent, review the Clinician Dashboard during the assigned intervention period, and complete surveys. In addition, interested clinics must agree to be randomized to the Clinician Dashboard intervention in the hybrid stepped wedge design (see Fig. 2). The study team will deploy a site screening survey in order to compile a list of interested surgical practices, including questions around practice type, new breast cancer patient volume, engagement of site clinicians, geographic diversity, and ability to enroll patients from minority and/or Spanish speaking patient populations, and history of performance on other NCORP CCDR trials. As sites complete the survey and indicate interest in joining SharES, a list will be compiled and from that a representative group of 25 surgical practices selected to represent geographic diversity, Spanish-speaking patient populations, and rural-urban variation. Each surgical clinic will receive $1000 as an appreciation of their participation in SharES, paid from the funded grant.

#### Clinicians

Clinician recruitment is concurrent with surgical clinician recruitment, since clinics cannot join the study without at least one committed breast surgeon. Clinicians must agree to utilize the Clinician Dashboard at the time period their practice is randomized to it. Once a surgeon/surgeons at each interested practice is identified, s/he will be rostered with the NCI (if not already), consented and registered. Where possible, participating clinicians will receive a $50 e-gift card for participating.

#### Patients

Eligible patients will be recruited by CRAs and staff at each of the 25 participating surgical clinics. SharES has developed a flexible approach to patient recruitment whereby patients may be recruited before or after their first surgical consult regarding their new diagnosis of breast cancer. This allows the participating clinics and clinicians to determine what works best for their practice; some surgeons prefer to see the patient first and determine whether she is truly eligible to consider surgical treatment options or whether she may need to consider only one option for clinical indication reasons, while other surgeons prefer patients to review patient facing material prior to meeting with them to discuss at the first consult. Patients may be recruited and enrolled in person or remotely using remote consent. Patients will receive a $20 e-gift card following registration and another $20 e-gift card following completion of each follow-up assessment.

## Assignment of interventions: allocation/randomization

### Sequence generation {16a}

Both clinics and patients will be randomized; cluster randomization for clinics in a hybrid stepped wedge fashion and individual randomization for patients is described below.

#### Clinic-level randomization

Twenty-five clinics will be identified for participation. Prior to study activation, clinics will be randomized to their respective time period (or “step”). Five clinics will be randomly allocated to receive the Clinician Dashboard for the entire duration of the study and five clinics will be randomly allocated to control with no Clinician Dashboard. Groups of three clinics will be randomly allocated to one of the five steps that have the Clinician Dashboard activated at a later time period (i.e., T2, T3, T4, T5, T6) (Fig. 2). Clinic-level randomization will not be stratified.

#### Patient-level randomization

Patient will be individually randomized in a 1:1 ratio to iCanDecide-S vs. iCanDecide-ESE using a dynamic allocation algorithm based on methods by Pocock and Simon [30]. The goal of the algorithm is to maintain arm balance with respect to the following stratification factors which are prognostic factors on study outcomes, especially knowledge: race (non-Hispanic White; non-Hispanic African American; Other—including Hispanic) and education status (≤ high school; some college; college graduate or more). Patients are assigned to the arm that leads to more imbalance 10% of the time to ensure some randomness in the algorithm.

### Concealment mechanism {16b}

The patients are allocated to one of the two iCanDecide versions but which version of the intervention they receive is concealed to them. Clinics and clinicians are not aware to which version their patients are randomized. The clinics/clinicians are made aware of their assignment 2 weeks prior to their Clinician Dashboard step.

### Implementation {16c}

Following enrollment, patients will be randomized by the Alliance Statistics and Data Management Center (SDC) as described above. Their allocation assignment will be securely sent to the UM CHCR technology development team. The CHCR team will send the patient an email with a direct link to her assigned group. All twenty-five clinics will be randomized at study initiation into one of seven steps (including one that never utilizes the Clinician Dashboard, one that always uses the Clinician Dashboard and five that begin to use the Clinician Dashboard at different times).

## Assignment of intervention: blinding

### Who will be blinded {17a}

The Alliance SDC and CHCR technology team cannot be blinded to the trial arm to which patients are assigned, or to which clinics are randomized, as they are responsible for randomization and allocation. The study investigators (principal investigators and co-investigators) are blinded to the trial arm. Patients will be blinded to which of the iCanDecide websites is the intervention being tested (iCanDecide-ESE) and which is the control (iCanDecide-S). Clinics cannot be blinded because they will be aware roughly 2 weeks prior to when they are allocated to start the Clinician Dashboard to receive training.

### Procedure for unblinding if needed {17b}

There is no requirement for this.

## Data collection

### Plans for assessment and collection of outcomes {18a}

Data collection for the primary, secondary, and additional outcomes related to locoregional treatment decision making and communication will be done via patient survey at 5 weeks post-surgery (first follow-up assessment). This will be done via the Patient Cloud (“ePRO”) application that will be added to the participant’s smart device (iPhone or Android) at time of enrollment, with a paper survey as a backup option for those who desire it. Participants will be surveyed again 9 months after registration (second follow-up assessment) to assess measures related to systemic treatment decision-making and communication (via ePRO or paper option). At 9 months after registration, participant’s medical records will be reviewed by the CRA for treatment information (surgery performed, use of radiation therapy, chemotherapy treatment). Clinicians will complete the same survey three times throughout the study, at time of registration, and 9 and 18 months after registration.

### Plans to promote participant retention and complete follow-up {18b}

Patients will be reminded by the CRA/clinical staff to log in and complete assessments. Clinicians will be reminded by the CRA/clinical staff to complete their assessments. Patients and clinicians receive incentives following completion of assessments.

### Data management {19}

Data will be managed by the SDMC.

### Confidentiality {27}

Data are stored in secure and confidential software systems affiliated with the Alliance and NCORP.

### Plans for collection, laboratory evaluation, and storage of biological specimens for genetic or molecular analysis {33}

Not applicable for this study.

## Analysis

### Statistical methods for primary and secondary outcomes {20a}

We will follow an intention to treat approach [31], using all available data, regardless of group assignment or whether patients viewed the patient-facing decision tool. The data as collected will have a nested structure: patients nested within clinicians, within clinics and regions of the country. Descriptive analyses will summarize patient, clinician, and clinic characteristics.

#### Primary outcome analysis

As described, we are testing two interventions, one randomized at the individual level (𝑖) and one at the clinic level (𝑗), rolled out to clinics over six trial time periods (𝑘). The interventions are fully crossed (with intervention and control patients within both intervention and control clinics for every time period). The linear mixed model for the continuous primary outcome (𝑦) patient knowledge has been adapted to a factorial design by adding an individual-level intervention to the model for a hybrid stepped wedge design [32]: 𝑦_𝑖𝑗𝑘_ = 𝛽_0_ + 𝜃_1_𝑥_1𝑖𝑗𝑘_ + 𝜃_2_𝑥_2𝑗𝑘_ + 𝛽_𝑘_𝑡_𝑘_ + 𝑢_𝑗_ + 𝑒_𝑖𝑗𝑘_. Here, 𝑦_𝑖𝑗𝑘_ denotes the response corresponding to individual 𝑖 at time period 𝑘 from clinic 𝑗 (𝑖 = 1, … , 𝑚_𝑗𝑘_ ; 𝑗 = 1, … , 25; 𝑘 = 1, … , 6). The variable 𝑥_1𝑖𝑗𝑘_ (=0, 1) denotes the intervention status of patient 𝑖 enrolled in clinic 𝑗 at time period 𝑘. The variable 𝑥_2𝑗𝑘_ (=0, 1) denotes the intervention status of this patient’s clinic 𝑗 at the 𝑘𝑡ℎ time period. The variable 𝑡_𝑘_ (=0, 1) is the indicator for time period 𝑘 and the term 𝛽_𝑘_ represents the corresponding fixed categorical effect to model the underlying secular trend (𝑘 in 1, …, 5, 𝛽_6_ = 0 for identifiability). The random effect 𝑢_𝑗_ is a random intercept for clinic 𝑗 and represents the time invariant deviation of the 𝑗𝑡ℎ clinic from the population average. The term 𝑒_𝑖𝑗𝑘_ is the patient error term. The term 𝛽_0_ is the intercept. The terms 𝜃_1_ and 𝜃_2_ represent the intervention effect of iCanDecide-ESE and the intervention effect of the Clinician Dashboard, respectively. As one of the most effective ways to improve the statistical power in cluster randomized trials [32, 33], the two patient-level stratification factors (race; education status) as well as an indicator for Hispanic/Latina (yes; no) that are expected to be strongly associated with the outcome knowledge will be included in the model as supportive analysis. The model assumes that an underlying time effect is the same for each clinic. To allow for potential clinic-level fluctuations of the time effect related to the roll out of the Clinician Dashboard, the random effect (𝑢𝑡)_𝑘_ (i.e., a clinic effect interacting with time) will be included in sensitivity analysis. Additionally, sensitivity analyses will account for nesting within clinician.

#### Co-primary objective #1

The analysis effectively reduces to a typical multisite, patient-level RCT of iCanDecide-S vs. ESE [32]. We will compare the interventions on patient knowledge, obtained at the first follow-up (5 weeks) assessment post-surgery. The effect of the patient-level intervention will be quantified by the coefficient 𝜃_1._

#### Co-primary objective #2

The primary estimate of the effect of the Clinician Dashboard cluster-level intervention on the primary outcome knowledge obtained at the first follow-up (5 weeks) assessment is quantified by the coefficient 𝜃_2_ .

Although a single model is estimated to address the two co-primary objectives, a fixed-sequence testing strategy will be applied to control the Type I error rate at 0.05 across the two corresponding primary comparisons. The iCanDecide-ESE vs iCanDecide-S comparison will be tested first at the significance level *α* = 0.05. Provided that the result achieves statistical significance at the 0.05 level, the Clinician Dashboard vs no Dashboard will be tested at the 5% significance level.

#### Secondary outcome analysis

We believe the two key secondary outcomes (cancer worry, breast cancer self-efficacy) will provide supportive information about the effectiveness of the iCanDecide-ESE and Clinician Dashboard on the primary outcome of knowledge. A gatekeeping statistical strategy will be used to control the type I error rate at 0.05 across the primary and this key secondary family of outcomes such that when both effects on the primary outcome of knowledge are demonstrated, this family of key secondary outcomes will be examined and may contribute important supportive information about the effectiveness of the interventions. The secondary family of tests comprises four tests (two for each secondary outcome) that will be conducted and will be tested with the Holm procedure [29]. The linear mixed model described will be separately fit for each continuous outcome (cancer worry; self-efficacy). The self-efficacy (or cancer worry) effect of the individual-level intervention will be quantified by the coefficient 𝜃_1_. The estimate of the effect of the Clinician Dashboard cluster-level intervention on self-efficacy (or cancer worry) will be quantified by the coefficient 𝜃_2_.

### Interim analysis {21b}

There is no planned interim analyses.

### Additional analyses {20b}

Additional secondary outcomes (HCCQ, PACE, SDQ) will be explored following the primary analysis described above. Concordance is a binary outcome reflecting whether the treatment received was concordant or not with the patient’s emotional support measures so a logistic random effects model similar to that above will be used.

The clinician measures that are collected at three time points (upon registration, 9 and 18 months after registration) will be compared. The purpose is to assess whether scores in clinician reported ability to deliver PCC (using the two measures included in their survey) increase as their clinic is exposed to using the Clinician Dashboard. There is no formal statistical testing planned for analysis of these measures.

### Methods to handle protocol non-adherence and statistical methods to handle missing data {20c}

This is an intent-to-treat analysis. We will measure our primary and key secondary outcomes by patient report in the first follow-up survey, 5 weeks post-surgery. We anticipate we will have minimal missingness at this time point based on our prior iCanDecide trial [3] for which we obtained 92% complete follow-up survey data at the same measurement time point. However, to decrease missing data from follow-up survey, assessment burden, and dropout, we have limited the number of survey questions and provided incentives. We have included flexible means of contacting patients, including email (primary modality), telephone, or regular mail. If data are missing on predictor variables of interest, data will be imputed using multiple imputation (MI) techniques (MI using chained equations) [34]. MI produces valid statistical estimates under missingness at random (MAR), while also accounting for additional uncertainty arising from missing data. We will perform sensitivity analyses to determine the degree to which results are sensitive to the MAR assumption. We will also account for survey non-response using an inverse probability weighting approach if we should find that there is differential survey non-response between the intervention and usual care groups according to patient characteristics that also influence the outcomes of interest. We will consider patients as participants if they complete enrollment and the baseline tailoring survey and only consider them to have dropped out if they withdraw consent. If a participant withdraws consent, we will document (1) the reason for dropout, (2) who decided the participant would drop out, and (3) whether the dropout involves some or all types of participation.

### Plans to give access to the full protocol, participant-level data, and statistical code {31c}

The full protocol resides with the NCI Central IRB and is available as a supplement. In accordance with the Alliance, data may be available upon request to the Principal Investigator.

## Oversight and monitoring

### Composition of the coordinating center {5d}

The study team includes the Principal Investigators and staff based at the coordinating center (University of Michigan) and those from the Alliance for Clinical Trials in Oncology cooperative group. The Alliance is led by a Group Chair who answers to a Board of Directors made up of clinical site principal site investigators from 50 Alliance member institutions. The vision for the Alliance is to reduce the impact of cancer by uniting a broad community of scientists and clinicians who are committed to the prevention and treatment of cancer.

### Composition of the data monitoring committee {21a}

The Alliance Data and Safety and Monitoring Board meets every 6 months (or more, if needed) and reviews the study progress. It is chaired by independent members. A report is submitted to the study Principal Investigators and team following each meeting.

### Adverse event reporting and harms {22}

This is a very low risk study of an educational intervention for patients and clinicians. Prior such studies have not generated any adverse events. However, should a participant feels distressed as a result of study participation, we encourage them to contact the Alliance study team as listed in the informed consent.

### Frequency and plans for auditing trial conduct {23}

The Alliance will monitor all aspects of the study on an ongoing basis.

### Plans for communicating important protocol amendments to relevant parties {25}

Changes to the protocol will follow the Alliance process for submission to the NCI Central IRB. Once approved, the existing protocol is updated, and participating clinics are made aware of updates via regular all-site meetings.

### Dissemination plans {31a}

The team will develop plans for publication and dissemination of results. This will follow the publications requirements of the Alliance Publications Committee. We will share all results with participating clinics, as well as with the Alliance CCDR Committee. We will work with the Alliance and participating sites to ensure patient participants are aware of publications resulting from this study.

## Discussion

Breast cancer patients face a challenging landscape of decisions that start at the time of diagnosis. A cancer diagnosis often triggers additional worry and distress that make it difficult for patients to make high quality decisions about their care. While decision support tools focused on the cognitive aspects of decision making exist, specifically helping to improve patients’ understanding of their disease and treatment options, there are none that also help to manage the emotional aspects of a cancer diagnosis. Doing so may help patient better accept and understand more cognitive information. Furthermore, while decision tools are often intended to support patient-provider communication and shared decision making, few existing tools “close the loop” by helping providers understand where patients may have deficits to decision making. Our study, an RCT of a Shared Decision Engagement System, specifically focuses on addressing both gaps in decision making intervention research by (1) helping support the emotional aspects of decision making by offering patients tools to manage worry and distress and support coping and (2) providing their surgeons with a dashboard that will help them review where the patient is with regard to her decision process and address any lingering deficits. Through this multilevel intervention trial, we aim to improve PCC in order to improve outcomes for newly diagnosed breast cancer patients.

## Trial status

Protocol version: 1

Date when recruitment was initiated: 1 March 2021

Date when recruitment will be completed (approximate): 1 September 2023

## Supplementary Information

**Additional file 1.** Model consent form for patients.

**Additional file 2.** Model informed consent for clinicians.

## Data Availability

Any data required to support the protocol can be supplied upon request.

## Abbreviations

CCDR: Cancer Care Delivery
CHCR: Center for Health Communications Research
CRA: Clinical Research Assistant
HCCQ: Healthcare Climate Questionnaire
NCI: National Cancer Institute
NCORP: NCI Oncology Research Program
PACE: Patient Assessment of Cancer Experiences
PCC: Patient-centered communication
RCT: Randomized controlled trial
SDC: Statistics and Data Center
SDQ: Subjective Decision Quality
SharES: Shared Decision Engagement System
