# Supplementary material for: Improving patient-centered communication in breast cancer: a study protocol for a multilevel intervention of a shared treatment deliberation system (SharES) within the NCI community oncology research program (NCORP) (Alliance A231901CD)
Source: Trials. 2023 Jan 6;24:16. doi: 10.1186/s13063-022-07048-4 (PMC9817354; doi:10.1186/s13063-022-07048-4)
Supplement: Supplementary file 1 — Additional file 1. Model consent form for patients. [file 13063_2022_7048_MOESM1_ESM.pdf]

MODEL CONSENT FORM FOR PATIENTS

Study Title for Participants: Improving Patient-Centered Communication in Breast Cancer through Patient and Clinician Interventions

Official Study Title for Internet Search on http://www.ClinicalTrials.gov: A231901CD, **"**Improving Patient-Centered Communication in Breast Cancer: A RCT of a Shared Decision Engagement System (SharES)**," (NCT04549571)**

Overview and Key Information

What am I being asked to do?

We are asking you to take part in a research study. This study has public funding from the National Cancer Institute (NCI), part of the National Institutes of Health (NIH) in the United States Department of Health and Human Services. We do research studies to try to answer questions about how to prevent, diagnose, and treat diseases like cancer.

We are asking you to take part in this research study because you have an appointment with your breast surgeon to discuss treatment for breast cancer, and have not yet received your surgical treatment.

Taking part in this study is your choice.

You can choose to take part or you can choose not to take part in this study. You also can change your mind at any time. Whatever choice you make, you will not lose access to your medical care or give up any legal rights or benefits.

This document has important information to help you make your choice. Take time to read it. Talk to the study staff, your family, or friends about the risks and benefits of taking part in the study. It's important that you have as much information as you need and that all your questions are answered. See the "Where can I get more information?" section for resources for more clinical trials and general cancer information.

Why is this study being done?

This study is being done to answer the following question:

How can we best help patients and their clinicians make decisions about breast cancer treatment (surgery and other treatments, like radiation and chemotherapy)?

We are doing this study because we want to better understand how to support patients in making decisions about breast cancer treatment.

We want to see if new approaches to support patient decision-making and patient-clinician communication about breast cancer treatment are better than existing or usual approaches. We are testing two interventions, one for patients and one for clinicians.

What is the usual approach to my diagnosis?

The usual approach for patients who are not in a study is to get advice about breast cancer treatment from their cancer-related doctors.

What are my choices if I decide not to take part in this study?

- You may choose to have the usual approach described above.
- You may choose to take part in a different research study, if one is available.

What will happen if I decide to take part in this study?

If you decide to take part in this study, you will:

- Be emailed a link to one of two decision tool websites that can be viewed on any computer or mobile device and asked to create an account. You can then view the website at your convenience as many times as you want before your surgery, and also after surgery if you are considering systemic treatment. Systemic treatment is treatment using substances that travel through the bloodstream, reaching and affecting cells all over the body, for example, chemotherapy.
- You will be asked to put a survey application, "App" on your mobile device to complete two surveys that will be sent to you about a month from your surgery date, and again about 9 months after you join the study (then, you can delete the App), or you could complete the surveys on paper.
- Agree that your surgeon (or their nurse or physician assistant) can have access to some of your responses to the website, like if you are very worried, or have questions about your treatment options. But if this makes you feel uncomfortable, you have the option not to make this information available to your surgeon.
- You will be asked at the end of this form for your consent to be contacted to participate in an optional one-on-one telephone interview. A few participants among those who agreed to be contacted will be selected by chance. If you are selected and contacted, you can decide at that time whether you want to participate in that interview.

After you finish participating in the study, you will continue with your usual care following breast cancer treatment as deemed necessary by your cancer-related doctors.

What are the risks and benefits of taking part in this study?

There are both risks and benefits to taking part in this study. It is important for you to think carefully about these as you make your decision.

Risks

You may have the following discomforts:

- Be asked sensitive or private questions about things you normally do not discuss.

More specifically:

- You may feel worried knowing that your responses to the website may be seen by your surgeon or their staff (nurse or physician assistant). This is done so your surgeon can help make sure all your questions are answered. But if this makes you feel uncomfortable, you have the option not to make this information available to your surgeon.
- It is also possible that you may have some anxiety from answering questions about your treatment decision during the surveys that will be done in the App. We recognize that this is a sensitive subject and some people would prefer not to talk about these issues. Your answers help us to understand if the websites worked to help with decision-making. But you may skip any questions on the surveys that you feel uncomfortable answering.
- There is a small risk that your study information could become known to someone who is not involved in performing or monitoring this study. However, we make every effort to protect your privacy. Our team follows rigorous standards to keep your information protected.

There may be some risks that the study doctors do not yet know about.

Benefits

You are not expected to have any direct medical benefit from participating in this study. However, there may be other benefits:

- Viewing information on the website may help you with your breast cancer treatment decision-making.
- Allowing your surgeon to view your responses may help make sure all your questions are answered.
- This study may help the study doctors learn things that may help other breast cancer patients in the future.

If I decide to take part in this study, can I stop later?

Yes, you can decide to stop taking part in the study at any time.

If you decide to stop, let your study doctor know as soon as possible. You also may choose to stop participation or skip any questions in the surveys that you do not feel comfortable answering.

Your study doctor will tell you about new information or changes in the study that may affect your health or your willingness to continue in the study.

Are there other reasons why I might stop being in the study?

Yes. The study doctor may take you off the study if:

- The study is stopped by the National Cancer Institute (NCI), Institutional Review Board (IRB), or study sponsor (the Alliance). The study sponsor is the organization who oversees the study.
- Your health changes and the study is no longer in your best interest.

This study is conducted by the Alliance for Clinical Trials in Oncology, a national clinical research group supported by the National Cancer Institute. The Alliance is made up of cancer doctors, health professionals, and laboratory researchers, whose goal is to develop better treatments for cancer, to prevent cancer, to reduce side effects from cancer, and to improve the quality of life of cancer patients.

It is important that you understand the information in the informed consent before making your decision. Please read, or have someone read to you, the rest of this document. If there is anything you don't understand, be sure to ask your study staff or nurse.

What is the purpose of this study?

The purpose of this study is to see if new approaches to support patient decision-making and patient-clinician communication about breast cancer treatment are better than existing or usual approaches. We are testing two interventions, one for patients and one for clinicians. The clinician could be your breast surgeon , nurse, physician assistants, nurse practitioner or clinical nurse specialist at your hospital who is engaged in your breast cancer care.

We will compare two different decision tool websites providing patients information about breast cancer treatment decisions and to help improve communications between patients and clinicians. The patient intervention will be used to help people become involved in decision-making by making clear the decision that needs to be made, providing information about the options and end results, and by explaining personal values. We will also compare an intervention for clinicians that helps them learn about ongoing needs after their patients use the decision tool websites, to usual care (no clinician intervention). The clinician intervention is a website for clinicians that will show them patient knowledge and values and their emotional state.

There will be about 700 patients taking part in this study. The study will enroll from 25 surgical practices in the USA and it is expected that enrollment will be a total of 75 clinicians.

This study will help the study doctors find out if one approach is better or the same as the other approach. To decide if it is better, the study doctors will be looking to see if knowledge about treatments are better or the same after patients look at each online website and after their clinicians use the clinician intervention.

What are the study groups?

This study has 2 study groups. You will not be told which group you are in.

All patients in this study will receive the usual approach of care, which is to get advice about breast cancer treatment from their doctor. Once you are enrolled to the study and provide your email address, you will also have access to one of two websites with high quality information about breast cancer treatment.

- **Group 1**

  If you are in this group, you will be sent an activation link to access a version of the website which includes information about breast cancer treatment that has been proven to help patients make their breast cancer treatment decisions. You will be asked to answer some questions on this website. The website will provide your surgeon (or your nurse or physician assistant) with access to your responses to the website. You will then be asked to complete two surveys: about one month after your surgery date, and again 9 months after you join the study. You will also be asked if you would like to participate in an optional interview at the end of the study, which will be audio recorded.

  There will be about 350 people in this group.

- **Group 2**

  If you are in this group, you will be sent an activation link to access a different version of the website which includes more information about managing breast cancer treatment than the one proven to help patients make their breast cancer treatment decisions. You will be asked to answer some questions on the website. The website will provide your surgeon (or your nurse or physician assistant) with access to your responses to the website. You will then be asked to complete two surveys: about one month after your surgery date , and again 9 months after you join the study. You will also be asked if you would like to participate in an optional interview at the end of the study, which will be audio recorded.

  There will be about 350 people in this group.

We will use a computer to assign you to one of the study groups. This process is called “randomization.” It means that your doctor will not choose and you cannot choose which study group you are in. You will be put into a group by chance. You will have an equal chance of being in Group 1 or Group 2.

Another way to find out what will happen to you during this study is to read the chart below. Start reading at the left side and read across to the right, following the lines and arrows.

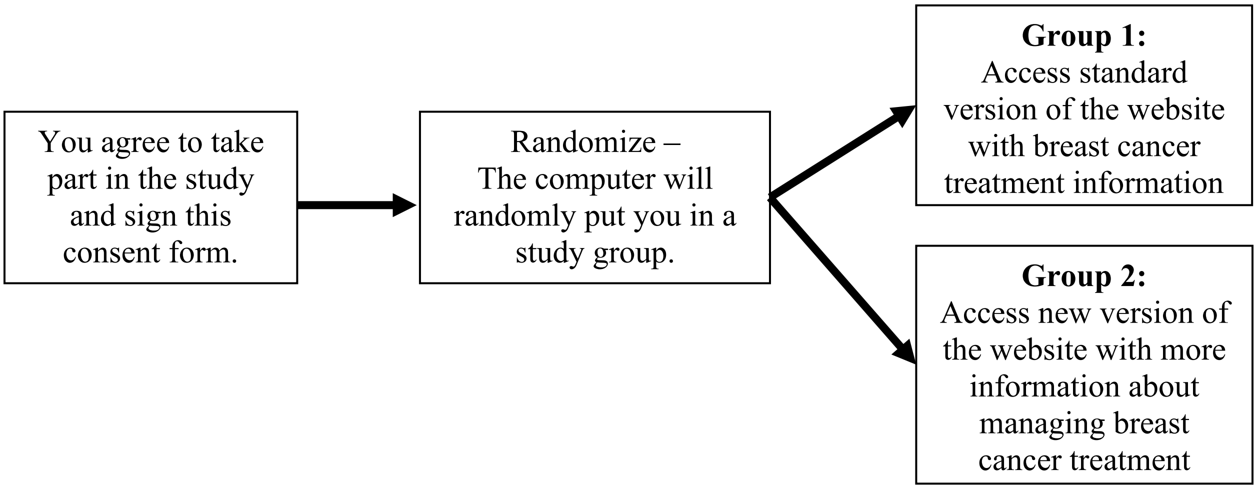

What procedures are involved in this study?

If you speak English and/or Spanish and decide to take part in this study, you will:

- Be emailed a link to one of the two websites in your language of choice that can be viewed on any computer or mobile device and asked to create an account. You can then view the website at your convenience as many times as you want before your surgery, and also after surgery if you are considering systemic treatment. The website may be used on a laptop, home computer or mobile device. If you do not have access to these, it may be viewed on a clinic iPad provided by the study. It may also be viewed on a device owned by a friend or family member, or in a public library.
- Agree that your surgeon (or their nurse or physician assistant) can have access to some of your responses to the website, like if you are very worried, or have questions about your treatment options. But if this makes you feel uncomfortable, you have the option not to make this information available to your surgeon.
- Agree that study staff will review your medical chart. We are doing this only to collect:
    - Demographic information (such as age)
    - Information related to the evaluation and treatment of breast cancer
- Be asked to complete two surveys with questions about how you feel about your treatment decision and communication with your surgeon and other cancer doctors, that will be sent to you about a month from your surgery date, and again about 9 months after you join the study. Each survey takes about 20-25 minutes to complete. Researchers will use this information to better understand how to support patients in making the decision about breast cancer surgery.

  Since these forms are being used for research, the responses you provide will not be shared with your study doctor. If you have any serious health issues or other concerns, please talk with your doctor or nurse right away.

Electronic surveys: For this study, you will be asked to complete the surveys stated above, on your personal smartphone or electronic device, which can be used to enter your answers to the questions. If you need help installing and/or using the survey application (or "app") on your phone or tablet, ask for help at your study site. Someone may help you enter your answers in the device if you need assistance. The use of your own electronic device on a cellular network may result in a small cost to your data plan. Regardless of the device you use, your answers and personal information will not be stored on the device.

Your survey answers will be sent to the research database and will be kept private as described in the section below called, "Who will see my medical information?" Your e-mail address will only be used for this survey and will not be used for mail or marketing purposes. The Alliance will not keep your email address.

If using your phone or a tablet is not possible or if you prefer to complete the surveys on paper, a paper survey will be provided by staff at your clinic, along with stamped addressed envelopes. You don't have to answer any question that makes you feel uncomfortable. You may mail these surveys or return them to the clinic at your next visit, within 2 months from the time they are completed. If you will be using your phone or tablet to complete your surveys, you will be asked to enter the date of your surgery on your device, so that the survey will become available on your device at the correct time.

Some study participants will be contacted at the very end of the study and asked to participate in an optional one-on-one interview. You can decide at that time whether you want to participate in that interview.

What are my responsibilities in this study?

If you choose to take part in this study you will need to:

- View a website at your convenience before surgery (and after surgery if you are considering systemic treatment).
- Complete two 20-25 minute surveys on a device or on paper.
    - About 1 or 2 months month after enrolling
    - About 9 months after enrolling (using the App on your mobile device, *OR* by mail if needed)

What are the costs of taking part in this study?

There is no medical care or testing being given as part of this study. You and/or your insurance plan will need to pay for the costs of medical care.

There are no expected costs to you for taking part in this study. Taking part in this study should not require you to do anything differently than you would for your usual breast cancer treatment. It is possible that you may want to make more visits or contact your doctors more than if you were getting the usual approach to treat your cancer. If so, you might:

- Have more travel costs.

- Need to take more time off work.
- Have other additional personal costs.

After enrolling in the study, and after you complete each survey, you will receive electronic gift cards of $20 each, in appreciation of your time. The first will be emailed after you create an account on the website. The second gift card will be emailed after you do your first survey, and the third gift card will be emailed after you do your second survey.

If you participate in the optional interviews after the second survey, you will be emailed another $20 gift card.

The maximum compensation for participation in this study is $80. All of the gift cards for this study will be sent by the team at University of Michigan.

Who will see my medical information?

Your privacy is very important to us. The study doctors will make every effort to protect it. The study doctors have a privacy permit to help protect your records if there is a court case . However, some of your medical information may be given out if required by law. If this should happen, the study doctors will do their best to make sure that any information that goes out to others will not identify who you are.

Some of your health information, such as information related to the evaluation and treatment of your breast cancer, will be kept by the study sponsor in a central research database. If information from this study is published or presented at scientific meetings, your name and other personal information will not be used.

In order to be in the study and to get access to the website, your email address will be collected when you enroll and provided to the University of Michigan study team. This email will be kept on a protected and secure database and will not be shared for any purpose. The date of your surgery once this is known, along with your patient ID will be sent to the University of Michigan study team, so that they may send you friendly reminders to complete the study surveys.
In order for study researchers at the University of Michigan to be able to contact you about possibly participating in the telephone or in-person interviews, it may be necessary for them to collect your name and phone number in addition to your email. In addition, if you choose to complete the follow up survey by mail, we will need your mailing address so that we can send it to you. Any such contact information will be kept only for the duration of the study.

There are organizations that may look at or receive copies of some of the information in your study records. Your health information in the research database also may be shared with these organizations. They must keep your information private, unless required by law to give it to another group.

Some of these organizations are:

- The Alliance for Clinical Trials in Oncology.

- The NCI Central IRB, which is a group of people who review the research with the goal of protecting the people who take part in the study.
- The NCI and the groups it works with to review research.

In addition to storing data in the study database, data from studies that are publicly funded may also be shared broadly for future research with protections for your privacy. The goal of this data sharing is to make more research possible that may improve people's health. Your study records may be stored and shared for future use in public databases. However, your name and other personal information will not be used.

Some types of future research may include looking at your information and information from other patients to see who had side effects across many studies or comparing new study data with older study data. However, right now we don't know what research may be done in the future using your information. This means that:

- You will not be asked if you agree to take part in the specific future research studies using your health information.
- You and your study doctor will not be told when or what type of research will be done.
- You will not get reports or other information about any research that is done using your information.

Where can I get more information?

You may visit the NCI web site at http://cancer.gov/ for more information about studies or general information about cancer. You may also call the NCI Cancer Information Service to get the same information at: 1-800-4-CANCER (1-800-422-6237).

A description of this clinical trial will be available on http://www.ClinicalTrials.gov, as required by U.S. Law. This Web site will not include information that can identify you. At most, the Web site will include a summary of the results. You can search this Web site at any time.

You can talk to the study staff about any questions or concerns you have about this study. Contact the study staff (*insert name of study staff*) at (*insert telephone number, and email address if appropriate*).

For questions about your rights while in this study, call the (*insert name of organization or center*) Institutional Review Board at (*insert telephone number*).

Optional studies that you can choose to take part in

This part of the consent form is about optional studies that you can choose to take part in. They are separate from the main study described above. This optional study will not benefit your health. The researchers leading this optional study hope to know your views about the study.

Taking part in this optional study is your choice. You can still take part in the main study even if you say "no" to this study. There is no penalty for saying "no". You and your insurance company will not be billed for this study.

Optional 30-minute interview

At the end of the main study, after viewing the website/online tool, the study doctors are interested in speaking with some participants over the telephone to hear more about their views about the study. This optional interview will take up to 30 minutes and will be audio-recorded. You don't have to answer any question that makes you feel uncomfortable. The recording will be transcribed and your name will not be included. A random sample of participants who choose to take part in this study will be selected for the interview.

Please circle your answer: I give the study team permission to contact me at the end of the study to discuss the possibility of participating in an optional audio-recorded interview.

YES NO

My signature agreeing to take part in the study

I have read this consent form or had it read to me. I have discussed it with the study doctor and my questions have been answered. I will be given a signed and dated copy of this form. I agree to take part in the main study.

Participant's signature

Date of signature

Signature of person(s) conducting the informed consent discussion

Date of signature
