# Supplementary material for: Improving patient-centered communication in breast cancer: a study protocol for a multilevel intervention of a shared treatment deliberation system (SharES) within the NCI community oncology research program (NCORP) (Alliance A231901CD)
Source: Trials. 2023 Jan 6;24:16. doi: 10.1186/s13063-022-07048-4 (PMC9817354; doi:10.1186/s13063-022-07048-4)
Supplement: Supplementary file 2 — Additional file 2. Model informed consent for clinicians. [file 13063_2022_7048_MOESM2_ESM.pdf]

MODEL INFORMED CONSENT FOR CLINICIANS

Study Title for Participants: Improving Patient-Centered Communication in Breast Cancer through Patient and Clinician Interventions

Official Study Title for Internet Search on http://www.ClinicalTrials.gov: A231901CD, "Improving Patient-Centered Communication in Breast Cancer: A RCT of a Shared Decision Engagement System (SharES)," **(NCT04549571)**

What am I being asked to do?

We are asking you to take part in a research study. This study has public funding from the National Cancer Institute (NCI), part of the National Institutes of Health (NIH) in the United States Department of Health and Human Services.You are invited to participate because you are a clinician who routinely cares for patients newly diagnosed with breast cancer as a surgical oncologist or advanced practice provider such as a physician assistant or nurse practitioner. This study will enroll from 25 surgical clinics. It is anticipated to enroll a total of 25-100 surgeons, nurses or physician assistants (if they work directly with a surgeon), 700 patients across multiple sites in the United States.

This study is conducted by the Alliance for Clinical Trials in Oncology, a national clinical research group supported by the National Cancer Institute. The Alliance is made up of cancer doctors, health professionals, and laboratory researchers, whose goal is to develop better treatments for cancer, to prevent cancer, to reduce side effects from cancer, and to improve the quality of life of cancer patients.

Taking part in this study is your choice.

Your participation is completely voluntary. If you decide not to participate or to withdraw from the study, it will have no effect on any services or treatment you are currently giving or your professional standing.

Why is this study being done?

This study is being done to answer the following question :

Can we test the effectiveness of two interventions on patient reported outcomes of patient centered communication and decision-making about breast cancer treatment?

The first intervention consists of enhancements to an existing patient-facing breast cancer treatment decision tool (called iCanDecide) that will support management of worry, distress and anxiety. The enhanced tool will be compared to the existing tool. The second intervention consists of a clinician dashboard that will populate information after patients view either website

regarding any ongoing issues or concerns (e.g, ongoing knowledge deficits, values mis-aligned with treatment intention).

What are my choices if I decide not to take part in this study?

- You may choose to take part in a different research study, if one is available.

What will happen if I decide to take part in this study?

If you decide to participate in this research study, you will be asked to participate in an initial assessment meeting in person or virtually to identify possible barriers and facilitators of the implementation of the decision aid. This assessment will be conducted by the Alliance study chair and/or co-chairs, or members of the University of Michigan project management team. You will be asked to share your DOB, race and ethnicity at time of enrollment to the study. You will be asked to complete one baseline survey before patient enrollment begins, and within two weeks after your enrollment in the study. This survey will ask about your attitudes towards patient-physician communication and shared decision-making. The initial assessment meeting should not take more than 15 minutes.

At a certain time point in the study, you and your clinic will then be asked to move into the clinician-facing intervention arm. Once in the intervention group, you will be trained by the study team, to use a clinical dashboard (CDB) which is an online platform through which you can see your patients' lingering knowledge deficits and planned treatment, including the emotional state of the patients. You will be asked to create a secure password protected account to access the CDB. If you are a surgeon, you may designate an advanced practice provider who works with you and cares for the patient to access the CDB as well. We will send all your new patients who will be presenting for an appointment to discuss treatment decisions our intervention, one of two breast cancer surgeon decision aids, depending on which arm they are assigned, if they are enrolled on this trial. Those patients will also complete one baseline tailoring survey, which includes a brief question set to tailor content on the intervention website. They will also be asked to complete two short follow up surveys about knowledge, worry and self-efficacy and possibly participate in an additional telephone interview. A random sample of patients who provided their consent to participate in the interview and be audio recorded will be contacted for a process evaluation.

The determination of when you and your clinic will move over to the intervention group will be randomized. Please note that once you cross over to the decision aid intervention arm, your role in the study will not change and you will continue with your usual practice for seeing patients. In addition, you will complete a survey 9 months and 18 months after study activation, regardless of when your site entered the intervention period. At the end of the study, you will be asked to participate in an optional process evaluation interview, which will be audio recorded. We anticipate enrolling 4 patients per clinic per time period (each time period is approximately 3 months) for a total of about 28 of your patients throughout an 18 month study period. If you are the only clinician participating from your clinic, these will all be your patients, otherwise they will be divided among participating surgeons and advanced practice providers.

At the start of the study, patients will be enrolled at all 25 participating clinics and randomized to one of the two decision tool websites. Regardless of when your clinic enters the intervention period, your patients will have access to and use the websites. Regardless of which website the patient is randomized to, the clinician dashboard will populate during the time period that that clinic is randomized to that intervention.
When the clinician dashboard is populated, you will receive an alert via email. You may then log in to view the responses of your patient following her use of the website. You may choose to reach out to her should there be a need to clarify anything but this is optional.

Your participation will require about 2 additional hours for participation in the implementation activities (about 1 hour), viewing of the clinician dashboard (a few minutes each time), surveys (about 10-15 minutes) and the audio-recordings, should you choose to participate in that phase of the study.

If you agree to be contacted to participate in an optional audio-recorded telephone interview at the end of the study, a random sample of participants who consented will be contacted at the very end of the study after we have finished the procedures described in this consent form . You can decide at that time whether you want to participate in the interview. Please see the “Optional Studies” section below for more information.

What are the risks of taking part in this study?

There is a risk that your study information could become known to someone who is not involved in performing or monitoring this study.

It is also possible that you may feel anxious from viewing information from your patient, for instance if her values are not aligned with her planned treatment. You will have the chance to follow up with her in this case.

If there are any patients you feel should not participate in the study, you will have the opportunity to tell study staff to exclude them from the study and they will not be approached by study staff.

Since it is possible that your patients may develop some anxiety or clinical questions from their own participation in the study (viewing the patient-facing website), there is a chance that they will want to speak with you or a member of your care team after a study survey or interview. This may result in additional time required from you to address their questions or concerns. If this requires an office visit or phone call, you will not be able to charge a fee if it is covered under the global payment for surgery.

What are the benefits of taking part in this study?

You are not expected to benefit directly from participating in this study. Your participation in this research study may benefit other people in the future by helping us learn more about breast cancer surgery decision-making between clinicians and patients.

Will I be paid for my participation?

You will receive a $50 electronic gift card in appreciation of your time. This will be emailed after you complete the first survey.

If you participate in the individual telephone interview, you will receive another $50 electronic gift card that will be emailed after your interview.

However, depending on local laws or institutional policies, you may not be able to receive your incentive, and we may not able to direct it to another source.

How will my confidentiality be protected?

Your privacy is important to us. Your survey responses will only include a study ID and will not include any personal identifying information. When you are enrolled to the study, your name and study ID will be sent to the University of Michigan study team, so that they can enable your access to the dashboard. They will also send you friendly reminders to complete the study surveys.

Conversations that are recorded throughout your participation (if you participate in the optional telephone interview at the end of the study) will be transcribed and coded with a study ID number. Only study staff and trained transcriptionists will have access to the audio files. All identifying information will be redacted in the written transcript. The audio files will be destroyed once the study is complete. Retained data (transcripts and surveys) will be de-identified once data collection is complete.

While there will likely be publications as a result of this study, your name will not be used. Only group characteristics will be published.

If you participate in this study, we would like to be able to quote the words you have used without using your name. If you agree to allow us to quote you in publications, please initial the statement at the bottom of this form.

A description of this clinical trial will be available on http://www.ClinicalTrials.gov, as required by U.S. Law. This Web site will not include information that can identify you. At most, the Web site will include a summary of the results. You can search this Web site at any time.

What if I have questions or concerns?

If you have any questions about this study at any time, contact the site Principal Investigator [PI NAME] at [PI PHONE NUMBER].

If you are not satisfied with the response of the research team, have more questions, or want to talk with someone about your rights as a research participant, contact the [SITE PATIENT RELATIONS REPRESENTATIVE] at [PHONE NUMBER].

Optional studies that you can choose to take part in

This part of the consent form is about optional studies that you can choose to take part in. They are separate from the main study described above. This optional study will not benefit you. The researchers leading this optional study hope to know your views about the study.

Taking part in this optional study is your choice. You can still take part in the main study even if you say "no" to this study. There is no penalty for saying "no".

Optional 30-minute interview

At the end of the main study, the study team will be interested in speaking with some participants over the telephone to hear more about their views about the study. This optional interview will take up to 30 minutes and will be audio-recorded. You don't have to answer any question that makes you feel uncomfortable. The recording will be transcribed and your name will not be included.

Please circle your answer: I give the study team permission to contact me at the end of the study to discuss the possibility of participating in an optional audio-recorded interview.

YES NO

Your signature indicates that you have read this consent form, had an opportunity to ask any questions about your participation in this research and voluntarily consent to participate. You will receive a copy of this form for your records.

This is the end of the section about optional studies.

My signature agreeing to take part in the study

I have read this consent form. I will be given a signed and dated copy of this form. I agree to take part in the main study. I also agree to take part in any additional studies where I circled "yes".

Participant Name (please print):____

Signature of Participant Date

I give my permission to be quoted directly in publications without using my name.
Initials

YOU WILL RECEIVE A COPY OF THIS FORM AFTER SIGNING IT.

Signature of person obtaining consent:

Signature Date
